# Meludia platform as a tool to evaluate music perception in pediatric and adult cochlear implant users

**DOI:** 10.1007/s00405-023-08121-7

**Published:** 2023-07-22

**Authors:** Miryam Calvino, Alejandro Zuazua, Isabel Sanchez-Cuadrado, Javier Gavilán, Marta Mancheño, Helena Arroyo, Luis Lassaletta

**Affiliations:** 1 Department of Otorhinolaryngology, Hospital Universitario La Paz. IdiPAZ Research Institute, Paseo de la Castellana 261, 28046 Madrid, Spain; 2 Biomedical Research Networking Centre on Rare Diseases (CIBERER), Institute of Health Carlos III (CIBERER-U761), Madrid, Spain; 3 Department of Otorhinolaryngology, Hospital Infanta Leonor, Madrid, Spain

**Keywords:** Cochlear implant, Audiology, Music, Rehabilitation, Cognitive training, Adolescent, Paediatric

## Abstract

**Purpose:**

Music perception is one of the greatest challenges for cochlear implant (CI) users. The aims of this study were: (i) to evaluate the music perception of CI users using the online Meludia music training program as music testing platform, (ii) to compare performance among three age groups, and (iii) to compare CI users with their normal hearing (NH) peers.

**Methods:**

138 individuals participated, divided between children (6–10 y), adolescents (11–16 y), and adults (≥ 17 y). Five music perception tasks were evaluated: Rhythm, Spatialization, Stable/unstable, Melody, and Density. We also administered the music related quality of life (MuRQoL) questionnaire for adults, and a music questionnaire for pediatric population (6–16 y) (MuQPP).

**Results:**

A significantly higher percentage of the adolescent CI users completed the five tasks compared to the other age groups. Both pediatric and adolescent CI users had similar performance to their NH peers in most categories. On the MuRQoL, adult NH listeners reported more music exposure than CI users (3.8 ± 0.6 vs 3.0 ± 0.6, *p* < 0.01), but both groups reported similar levels of perceived music importance (3.4 ± 0.7 vs 3.2 ± 1.1, *p* = 0.340). On the MuQPP, pediatric CI users who scored highly on music perception also had higher reported questionnaire scores (54.2 ± 12.9 vs 40.9 ± 12.1, *p* = 0.009).

**Conclusions:**

Meludia can be used to evaluate music perception and to use for music training in CI users of all ages. Adolescents had the highest performance in most musical tasks. Pediatric CI users were more similar to their NH peers. The importance of music in adult CI users was comparable to their NH peers.

**Supplementary Information:**

The online version contains supplementary material available at 10.1007/s00405-023-08121-7.

## Introduction

With modern cochlear implants (CI), users of all ages can obtain high levels of speech perception [1]. Music perception, however, is particularly challenging for CI users. This is due to biological limitations imposed by their hearing pathology and because spectral complexity of music relative to speech makes music reproduction challenging for CIs [2].

Users have different ways of coping with these deficits in music perception. Most children or adolescents with congenital or prelingual hearing loss find music positive and appealing [3], and often participate in musical activities [4]. Adult CI users, however, often avoid music because they find it unpleasant [5, 6].

As part of the broader rehabilitative program, training and evaluation programs for music perception have been developed [7–11]. Along with the enhancement of music perception itself, these programs can be a valuable tool in the assessment of the everyday benefit of CI use. Music training also enhances speech perception, reinforcing the benefits of CI use in other domains of hearing [12, 13].

The Meludia platform (Meludia, Paris, FR) is one such music training program. It is an online interactive program which employs progressive listening exercises to train music perception. Although Meludia was not designed specifically for CI rehabilitation, it has been used in previous CI research [10, 14]. It has been shown that the easiest Meludia exercises on the platform are accessible to adult CI users independently of age, pathology, duration of CI use, or musical background [14]. In a randomized controlled trial on adult CI users, Meludia training improved pitch discrimination and timbre identification [10], two aspects of sound perception that are difficult for untrained CI users.

To the best of our knowledge, Meludia has not been tested in pediatric CI users. It has not also been established whether the accessibility of the exercises, and the training benefits derived therefrom, translate to younger users. If they do translate, it is also not known how they compare between adult and pediatric users. The aims of the present study were therefore to: (i) evaluate different music tasks through the Meludia platform in experienced CI users, (ii) compare the results among three age cohorts, and (iii) compare the results with age-matched normal hearing (NH) peers.

## Methods

### Participants

This cross-sectional study took place between October’20 and October’22. We recruited two groups of participants: CI users and individuals with NH.

CI users included prelingually deafened patients who had received a CI before the age of 3 years, and aged between 6 and 16 years at the time of the study. They were divided in two groups, children: 6–10 y, and adolescents: 11–16 y. An additional group of postlingually deafened CI adults (≥ 17 y) was also included. All were uni- or bilaterally implanted with a MEDEL CI system (MED-EL GmbH, Innsbruck, Austria), had at least 10 active electrodes, and had at least 1 year of stable fitting.

Individuals with NH were also recruited to serve as control group. This group was selected to be age-matched with each of the three age cohorts of the CI users. Audiometric evaluation confirmed their status as NH listeners.

All participants were fluent in Spanish and were without concomitant visual or cognitive impairments that could interfere with completion of the tasks.

The study design was approved by the local Ethics Committee (approval number HULP PI-4447) and was registered at ClinicalTrials.gov (identifier NCT05319678). No adverse events were reported during the course of the study.

### Meludia

For this study, the Discovery module of Meludia was used. This module assesses five tasks, each with five levels of difficulty, giving a total of 25 exercises [15]:

- Rhythm: How many percussive hits do you hear?
- Spatialization: Is the second note higher or lower?
- Melody: Is the melody ascending or descending?
- Stable/Unstable: Does the sound feel stable or unstable?
- Density: How many sounds are played simultaneously? One or many?

Prior to the evaluation, a demonstration of the program was given to all participants in a quiet environment. NH subjects were tested using headphones connected to a laptop. For CI users, the direct audio input (DAI) cable was used to ensure that hearing was tested via CI alone, avoiding hearing through the contralateral ear in the case of residual hearing. Bilaterally implanted participants were tested with both implants simultaneously, i.e., bilaterally.

For each exercise, Meludia outputs a numeric score from 0–3, where 0 indicates that the attempt was incomplete and 3 indicates the fastest and most precise performance on the exercise. Listeners were permitted to restart each level up to a maximum of four times per level. If the exercise was not completed after four restarts, it was interpreted as incomplete and the program moved to the next exercise. The tasks were presented in the order: Rhythm, Spatialization, Melody, Stable/unstable, and Density. The average time to complete all these tasks was 60 min.

### Questionnaires

To evaluate musical background, two Spanish-language questionnaires were used: the Music related quality of life (MuRQoL) and the Music questionnaire for pediatric population (MuQPP) (see Supplementary material).

#### The MuRQoL

The MuRQoL assesses the impact of music on quality of life along two dimensions: frequency of music perception and engagement (questions 1–11) and musical importance (questions 12–18). The first part assesses subjective music perceptual abilities. The second part assesses the perceived importance of music perception and engagement attitudes towards music [16]. Responses are scored on a 5-point Likert scale (from 1: never to 5: always). Three scores were obtained for each participant: the total score, the music perception score, and the musical engagement score.

#### The MuQPP

After reviewing the existing literature, we did not find a questionnaire suitable both in language and in form for subjects aged 6–16 years. Therefore, we created a music questionnaire for pediatric population (MuQPP). It consists of questions that had already been validated and used by other authors [17–19] to assess musical interests, frequency items, role of music in daily life, and music-related activities.

### Statistical analysis

Demographic characteristics and outcome measures are shown as absolute numbers (n) and relative frequencies (%), and if appropriate with mean ± standard deviation (SD) and range. Each Meludia level was scored as either complete or incomplete. The mean (± SD) was also calculated.

The data were normally distributed according to the *Kolmogorov–Smirnov test.* Intergroup (CI and NH) and intragroup (pediatric and adult populations) comparisons were made via *Student’s T-tes*t. The Chi-Square was used to compare the numbers of participants who completed the levels of each Meludia category.

Pearson’s correlation coefficient was calculated to assess the relationship between mean score of the different categories of Meludia program, and the corresponding subscales of the MuRQoL and the MuQPP.

A *p* value of ≤ 0.05 (2-tailed) was considered significant. Statistical analyses were conducted with the SPSS software package v24.0 (IBM, Armonk, NY, USA).

## Results

Participants were sixty-nine CI users: 14 children, 16 adolescents, and 39 adults. The same numbers of age-matched NH listeners participated. The demographic characteristics of all groups are given in Table 1.

**Table 1:** Demographic information of the participants

	CI users			NH listeners		
	Children	Adolescents	Adults	Children	Adolescents	Adults
N	14	16	39	14	16	39
Male (n) (%)	4 (29%)	9 (56%)	17 (44%)	6 (43%)	8 (50%)	15 (39%)
Female (n) (%)	10 (71%)	7 (44%)	22 (56%)	8 (57%)	8 (50%)	24 (61%)
Age (years) ± SD (range)	8 ± 1 (6–10)	14 ± 1 (12–16)	56 ± 19 (17–86)	8 ± 1 (6–10)	13 ± 1 (11–16)	57 ± 13 (24–80)
CI duration* (years) ± SD (range)	7 ± 1 (5–9)	12 ± 1 (10–14)	8 ± 6 (1–19)	N/A	N/A	N/A
Type of implantation (n) (%)						
Unilateral	0 (0%)	1 (6%)	28 (72%)	N/A	N/A	N/A
Bilateral	13 (93%)	15 (94%)	2 (5%)	N/A	N/A	N/A
Bimodal	1 (7%)	0 (0%)	9 (23%)	N/A	N/A	N/A

Note that in case of sequential bilateral implantation, the duration since the first implantation is given

*SD* standard deviation; *N/A* not applicable

### Meludia task completion rates

#### CI users

For all tasks, a higher percentage of adolescent users completed the task at the fifth level than either children or adults. The greatest difference was with Melody, completed by 50% of adolescents, compared to 8% of adults (*p* < 0.001) and 14% of children (*p* = 0.038).

**Fig. 1:**
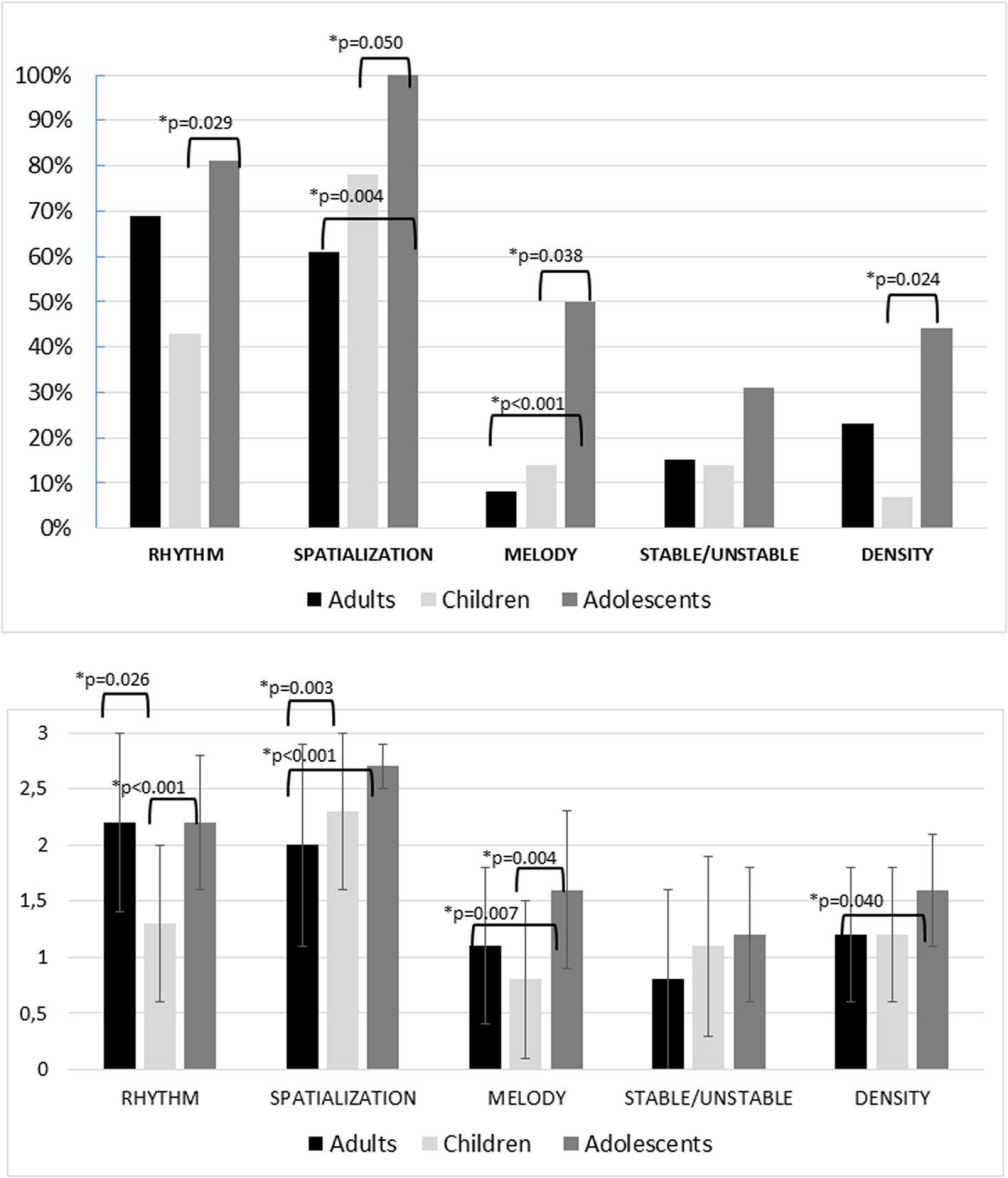
Top: Percent of CI users who completed the five levels of each task. Bottom: The mean scores and standard deviations of the three age groups of CI users on each task. Higher scores indicate better performance. *means *p* ≤ 0.05

There were no significant differences among the age groups of CI users in terms of the number of attempts needed to complete the tasks, with the exception of the Spatialization in which adult CI users needed more restarts than adolescents (6.1 ± 1.6 vs 5.2 ± 0.4, respectively, *p* = 0.002) (Fig. 1_top).

#### Comparison between CI users and NH peers

Amongst adults, the percentage of participants who completed each task was significantly higher for NH participants (all p < 0.05). In children, a significant difference was found only in Melody (*p* = 0.043). In the adolescent population, significantly greater percentages of NH listeners completed Stable/unstable (*p* = 0.001) and Density (*p* = 0.009) (See Table 2).

**Table 2:** Percentage of participants who completed the five levels of each task

	Adults			Children			Adolescents		
	CI users (%)	NH (%)	*p*	CI users (%)	NH (%)	*p*	CI users (%)	NH (%)	*p*
RHYTHM	69	90	0.025*	43	50	0.705	81	94	0.285
SPATIALIZATION	61	97	< 0.001*	78	93	0.280	100	100	–
MELODY	8	64	< 0.001*	14	50	0.043*	50	75	0.144
STABLE/UNSTABLE	15	59	< 0.001*	14	43	0.094	31	87	0.001*
DENSITY	23	61	0.001*	7	36	0.065	44	88	0.009*

*means *p* ≤ 0.05

Moreover, in Table 3 is displayed the percentage of participants who completed the first level of each task. Note that in all categories more than 70% of subjects passed the first level.

**Table 3:** Percentage of participants who completed the first level of each task

	Children		Adolescents		Adults	
	CI users (%)	NH (%)	CI users (%)	NH (%)	CI users (%)	NH (%)
Rhythm	93	93	100	100	97	100
Spatialization	100	100	100	100	90	100
Melody	71	93	100	100	95	95
Stable/unstable	86	85	93	100	74	95
Density	100	100	100	100	97	97

### Performance on Meludia tasks

#### CI users

Adolescents had the highest mean scores on all tasks. Adolescents significantly outperformed adults on Spatialization, Melody, and Density, and significantly outperformed children on the Rhythm and Melody. Adults significantly outperformed children on Melody, and children significantly outperformed adults on Spatialization. No significant differences among the age groups were observed for Stable/unstable (Fig. 1_bottom).

#### Comparison between CI users and NH peers

NH listeners generally obtained higher scores than CI users of the same age group. These differences were significant between the adult groups for all tasks. Among children, NH listeners did significantly better on Melody (*p* = 0.017). Among adolescents, NH listeners did significantly better on Stable/unstable (*p* < 0.001) and Density (*p* = 0.002) (Table 4).

**Table 4:** Mean scores ± standard deviation for all groups of participants for all tasks

	Children			Adolescents			Adults		
	CI users	NH	*p* value	CI users	NH	*p* value	CI users	NH	*p* value
Rhythm	1.3 ± 0.7	1.7 ± 1.1	0.196	2.2 ± 0.6	2.3 ± 0.6	0.781	2.2 ± 0.8	2.7 ± 0.4	0.007*
Spatialization	2.3 ± 0.7	2.5 ± 0.5	0.504	2.7 ± 0.2	2.6 ± 0.3	0.121	2.0 ± 0.9	2.6 ± 0.4	< 0.001*
Melody	0.8 ± 0.7	1.6 ± 1.0	0.017*	1.6 ± 0.7	2.0 ± 0.7	0.154	1.1 ± 0.7	1.9 ± 0.9	< 0.001*
Stable/unstable	1.1 ± 0.8	1.3 ± 0.9	0.454	1.2 ± 0.6	2.1 ± 0.5	< 0.001*	0.8 ± 0.8	1.6 ± 0.8	< 0.001*
Density	1.2 ± 0.6	1.4 ± 0.8	0.522	1.6 ± 0.5	2.3 ± 0.6	0.002*	1.2 ± 0.6	1.7 ± 0.8	0.004*

*means *p* ≤ 0.05

### Questionnaires

#### MuRQoL

**Fig. 2:**
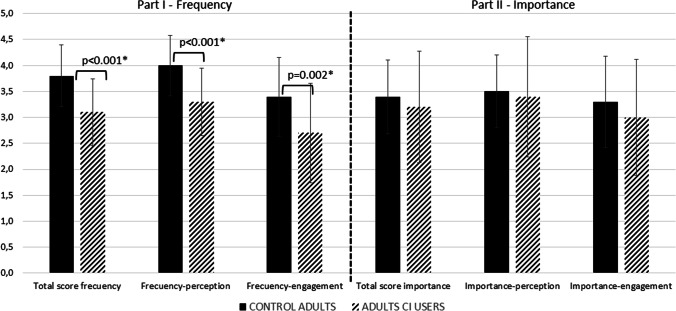
MuRQoL scores for both groups of adult participants. Left: Part I total score, frequency of music perception, and frequency of engagement. Right: Part II total score, importance of music perception, and importance of engagement. Bar charts represent means and standard deviation. *means *p* ≤ 0.05

Adult NH listeners had higher mean scores than CI users on the first part of the questionnaire (all *p* < 0.01) (Fig. 2). Both groups scored similarly in the second part, although the variance of scores was somewhat greater for the CI users.

#### MuQPP

Pediatric CI users had significantly higher mean scores than adolescent CI users (16.1 ± 3.8 vs 12.2 ± 5.1, *p* = 0.035). Moreover, 36% of pediatric CI users participated in music-related activities, while no adolescents did (*p* = 0.027).

Pediatric and adolescent CI users had similar scores to their NH peers on musical interests, musical profile and frequency items, and in the importance of music in their lives. The exception was pediatric CI users, whose mean score in their musical profile was significantly higher than their NH peers (54.2 ± 12.9 vs 40.9 ± 12.1, *p* = 0.009). More NH children participated in music-related activities than their CI user peers (50% vs 36%, *p* = 0.445). 40% of NH adolescents participated in musical activities, while none of their CI user peers did (*p* = 0.017).

#### Correlations between questionnaire responses and Meludia scores

In adult CI users, there was a significant positive correlation between the mean Meludia scores on Spatialization, Stable/unstable, Melody, and Density and scores on the MuRQoL part I. There was also a significant correlation between Rhythm scores and the MuRQoL part II.

In adult NH listeners, there was a significant positive correlation between the mean Meludia scores on Stable/unstable, Melody, and Density and the scores obtained in both parts of the MuRQoL (Table 5).

**Table 5:** Correlations between Meludia task scores and MuRQoL scores

	NH		CI users	
Rhythm				0.403
				0.020*
Spatialization			0.386	
			0.024*	
Stable/unstable	0.552	0.336	0.34	
	<0.001*	0.039*	0.049*	
Melody	0.498	0.321	0.361	
	0.001*	0.050*	0.036*	
Density	0.484	0.386	0.38	
	0.002*	0.017*	0.027*	
	Total score (frequency)	Total score (importance)	Total score (frequency)	Total score (importance)

Top: Pearson’s coefficient. Bottom: significance level

*means *p* ≤ 0.05

No significant correlations were found between the MuQPP responses and Meludia scores for pediatric or adolescent participants.

## Discussion

Music perception is the second most important motivating factor for CI users [20]. However, CI users have significant limitations in music perception. Nevertheless, the present state of CI technology, combined with the underlying hearing pathology, can prevent the full enjoyment of music. Although there is a large body of work dedicated to the evaluation of musical skills in CI users [21], there is no consensus on the best way of measuring music perception and music enjoyment in CI users.

In recent years, several music training programs for CI users have been introduced [7, 9, 11, 22, 23]. One such program, Meludia, has been validated for adult CI users [14, 24]. Here, we employed the online Meludia music training program as an evaluation tool in pediatric, adolescent, and adult CI users (range 6–86 years), demonstrating that Meludia provide good access for all ages, which means CI users can use Meludia to start music training with this program.

In the present study, adolescent CI users scored higher than adults and children in the Rhythm, Spatialization, Melody, and Density tasks. They also performed particularly well on Spatialization and had the highest completion rate (100%). One possible explanation for the higher performance of adolescents may be a combination of longer duration of CI use compared to children, while having a greater degree of neuroplasticity than adults, and especially elderly users. CI use can lead to neuroplastic changes within the auditory cortex [25].

Several studies have reported on music perception in adolescents [26–29]. The adolescent CI users in Driscoll et al. [26] showed greater music perception abilities than children. Similarly, Yüksel et al. [29] found that CI users aged 9–13 years with significant residual hearing had pitch discrimination scores similar to those of their NH peers. In another study, Yüksel el al. [28]. concluded that adolescents enjoy music to a similar degree as adult CI users. In contrast, the group of adolescent users in Jung et al. [27] had worse music perception than the adults, leading the authors to hypothesize this may be due to delayed maturation in temporal processing ability.

### Rhythm

Rhythm is considered a central element of music. Melodies can sometimes be recognized based upon rhythm alone. In CI users, rhythm perception is typically much better than pitch perception [30]. This is related to the fact that CI stimulation of the auditory nerve provides far more reliable temporal than spectral information. In our study, adults had the highest performance in the Meludia Rhythm task. Younger children often struggled with Rhythm because they had difficulty counting the percussive hits; indicating a deficit in the ability to count rather than a deficit in hearing perception.

### Spatialization

In the Spatialization task, the pediatric group had the highest performance scores. 100% of adolescent CI users and 78% of pediatric CI users were able to complete the most difficult level, which covers intervals as small as one semitone [14]. This contrasts with previous work which reported that pediatric CI users have a mean discrimination threshold of 2–3 semitones [27].

### Melody

Melody is a combination of pitch and rhythm [31]. In our study, CI users performed considerably worse at Melody than Rhythm. Only 8% of adult CI users completed fifth level of Melody. Boyer et al. [14] also found that adult CI users judged Melody as being difficult. Note that Melody exercises in Meludia do not have relevant rhythmic information, and also no reference tone; i.e., the first note of the ascending or descending melody starts at any pitch, which makes this task more challenging.

### Density

Density measures the ability of participants to detect whether one or many sounds are played simultaneously. This is related to the musical feature of harmony. In our study, only 7% of pediatric users completed the five levels of the Density task. There is evidence that in children, density/harmony perception develops slower than perception of other aspects of music [32]. Ab Shukor et al. [33] also found that pediatric CI users performed worse in harmony detection compared with other music features such as rhythm and pitch.

### Stable/unstable

Stable/unstable is the ability to recognize consonance and dissonance in musical sounds. Like density, it therefore also relies on the ability to detect multiple simultaneous sounds. This perceptual ability plays an important role in detecting musical emotion [34]. In our study, all age groups of CI users performed poorly on this task, and no significant differences were observed between the groups.

### Differences in music tasks: CI users vs NH participants

Among adult listeners, is generally reported that those with NH perform better than CI users on music perception tests. The present study is in agreement with this. Some studies [35, 36] have reported that CI users have access to rhythm perception within the NH range, although this was not borne out in our study; our CI users performed significantly poorer at this task.

Among the pediatric participants, CI users and NH listeners had similar scores in all the tasks, except for melody perception. In adolescents, NH listeners had better performance than CI users on Stable/unstable and Density. These differences between the pediatric and adolescent groups suggest that pediatric CI users may in some cases have greater access to musical perceptual abilities than adolescent users [37]. A recent review of 10 studies with 186 participants concluded that music training benefits are dependent upon age and duration of practice [38].

### Music listening habits

Another important point to consider is the music background of the participant, as well as the subjective evaluation of music enjoyment. Musical experience has been assessed in CI subjects, with heterogeneous results being reported [4, 5]. In 2007 our group [39] reported that 50% of CI users still enjoy music, despite a decrease in the quantity of music listened to.

In the present study, we found that both adult CI users and adult NH listeners give equal importance to music, despite NH listeners scoring higher in music listening frequency. These findings are in line with our previous results, and with other studies employing the same questionnaire [16, 40, 41].

Frosilini et al. [41] reported that NH amateur musicians had higher scores than non-musicians in terms of frequency and importance, and that even the non-musicians had higher scores than CI users. Surprisingly, Boyer et al. [14] concluded that music background had no effect on their outcomes either for CI users or NH participants. We consider it is important to point out they evaluated musical background/education only with a single question, according to three categories (none, initial and prolonged). Lassaletta et al. [6], using a different battery of tests, also found no difference in rhythm and tone scores between CI users who enjoyed or did not enjoy music after implantation.

### Impact of subjective music perception on Meludia scores

We assessed the correlations between CI users’ scores obtained by MuRQoL and their performance in a musical training program. Our findings show that the higher the frequency of music exposure, the better the scores were in Spatialization, Stable/unstable, Melody and Density. This was true of both CI users and NH listeners.

Regarding the results obtained with the MuQPP, it is interesting that scores on the frequency scale were higher in the pediatric CI users than the NH participants. Similar findings have been reported elsewhere, albeit with different questionnaires and somewhat different age ranges [4, 28].

It is also noteworthy that none of the adolescents CI users reported performing and music-related activity, while 40% of their NH peers did. In the Meludia tasks, we found that adolescents CI users had much poorer scores on Stable/unstable (related to consonance/dissonance perception) and Density (related to harmony perception). One explanation is that poor perceptual skills in these areas preclude either the acquisition of musical abilities with instruments or singing, or precludes the enjoyment derived from these activities that NH listeners obtain.

### Future directions, how to improve music perception in CI users

There are several potential ways to enhance music perception in CI users. These include improving coding strategies to process acoustic signals with high spectral complexity [42]; to improve surgical procedures in order to achieve maximum cochlear coverage with the most appropriate array length [42]; and to ensure that devices are fit not just with generic fitting algorithms, but tailored to each user’s anatomy and array positioning as identified by imaging [43]. Other methods to improve music perception include the integration of music training into the wider rehabilitative program [44] and to identify specific music characteristics that were more satisfying for CI users, and devise strategies to re-engineer music to emphasize these characteristics [45].

## Conclusions

Meludia is an appropriate tool to evaluate music perception in CI users and to use for music training, including younger children and older adults. Adolescents perform better than children and adult CI users in some musical tasks. In general, pediatric CI users are more similar to their NH peers than adults regarding musical perception. Subjectively, the importance of music in adult CI users was comparable to their NH peers.

### Supplementary Information

Below is the link to the electronic supplementary material.

Supplementary file1 (PDF 590 KB)

## Data Availability

The data presented in this study are available on request from the corresponding author.
