# Supplementary material for: Meludia platform as a tool to evaluate music perception in pediatric and adult cochlear implant users
Source: Eur Arch Otorhinolaryngol. 2023 Jul 22;281(2):629–38. doi: 10.1007/s00405-023-08121-7 (PMC10796694; doi:10.1007/s00405-023-08121-7)
Supplement: Supplementary file 1 — Supplementary file1 (PDF 590 KB) [file 405_2023_8121_MOESM1_ESM.pdf]

El Cuestionario de Calidad de Vida Relacionado con la Música

Primera parte

La primera parte del cuestionario incluye una serie de preguntas sobre su capacidad de escuchar música, su actitud hacia la música y sus actividades musicales. Por favor, conteste a las preguntas marcando una de las siguientes opciones: **1: nunca**, **2: pocas veces**, **3: a veces**, **4: con frecuencia**, **5: siempre**, **N/A: no aplicable**.

	1	2	3	4	5	N/A
**PERCEPCIÓN MUSICAL**						
1. ¿Puede distinguir distintos ritmos musicales?	☐	☐	☐	☐	☐	☐
2. ¿Puede seguir una melodía musical (es decir, seguir la melodía de una canción o de una pieza musical familiar)?	☐	☐	☐	☐	☐	☐
3. ¿Puede diferenciar tonos musicales (es decir, tonos agudos y graves)?	☐	☐	☐	☐	☐	☐
4. ¿Puede reconocer la letra de las canciones?	☐	☐	☐	☐	☐	☐
5. ¿Puede reconocer los sonidos de diferentes instrumentos musicales?	☐	☐	☐	☐	☐	☐
6. ¿Puede escuchar el significado de una pieza musical (es decir, la emoción, la razón por la que fue compuesta o el mensaje que intenta transmitir)?	☐	☐	☐	☐	☐	☐
7. ¿Puede escuchar música sin esfuerzo y sin la necesidad de concentrarse?	☐	☐	☐	☐	☐	☐
8. ¿Puede reconocer una melodía familiar (p.ej. una canción, un/a cantante, una pieza musical)?	☐	☐	☐	☐	☐	☐
9. ¿Puede valorar la calidad de una interpretación musical (p.ej. cuando alguien canta o toca un instrumento musical)?	☐	☐	☐	☐	☐	☐
10. ¿Tiene la sensación de que escucha música igual que otras personas?	☐	☐	☐	☐	☐	☐
11. ¿Le suena la música afinada?	☐	☐	☐	☐	☐	☐
**DEDICACIÓN A LA MÚSICA**						
12. ¿Disfruta de la música en entornos ruidosos si no hay referencias visuales (p.ej. en una fiesta, en un restaurante o en el coche con el ruido del motor o de la calle de fondo)?	☐	☐	☐	☐	☐	☐
13. ¿Disfruta de la música en la televisión, en el reproductor de DVD o en el ordenador?	☐	☐	☐	☐	☐	☐
14. ¿Pone usted música de fondo durante la práctica de actividades como la lectura, la pintura, la jardinería, el ejercicio físico, etc. o mientras se relaja?	☐	☐	☐	☐	☐	☐
15. ¿Escucha música al viajar (p.ej. en el coche)?	☐	☐	☐	☐	☐	☐
16. ¿Escucha música nueva (es decir, música que no ha escuchado antes)?	☐	☐	☐	☐	☐	☐
17. ¿Asiste a eventos musicales públicos (p.ej. musicales, conciertos o festivales de música)?	☐	☐	☐	☐	☐	☐
18. ¿Canta, toca algún instrumento musical o silba?	☐	☐	☐	☐	☐	☐

Segunda parte

La segunda parte del cuestionario evalúa la importancia que tiene para usted la capacidad de escuchar música, la actitud hacia la música y las actividades musicales descritas en la primera parte del cuestionario. Por favor, conteste a las preguntas marcando una de las siguientes opciones: **1: nada importante; 2: poco importante; 3: algo importante; 4: muy importante; 5: extremadamente importante, N/A: no aplicable.**

	1	2	3	4	5	N/A
**PERCEPCIÓN MUSICAL**						
1. ¿Qué importancia tiene para usted la capacidad de distinguir distintos ritmos musicales?	☐	☐	☐	☐	☐	☐
2. ¿Qué importancia tiene para usted la capacidad de seguir una melodía musical (es decir, seguir la melodía de una canción o de una pieza musical familiar)?	☐	☐	☐	☐	☐	☐
3. ¿Qué importancia tiene para usted la capacidad de diferenciar tonos musicales (es decir, agudos y graves)?	☐	☐	☐	☐	☐	☐
4. ¿Qué importancia tiene para usted la capacidad de reconocer la letra de las canciones?	☐	☐	☐	☐	☐	☐
5. ¿Qué importancia tiene para usted la capacidad de reconocer los sonidos de diferentes instrumentos musicales?	☐	☐	☐	☐	☐	☐
6. ¿Qué importancia tiene para usted la capacidad de escuchar el significado de una pieza musical (es decir, la emoción, la razón por la que fue compuesta o el mensaje que intenta transmitir)?	☐	☐	☐	☐	☐	☐
7. ¿Qué importancia tiene para usted la capacidad de escuchar música sin esfuerzo y sin la necesidad de concentrarse?	☐	☐	☐	☐	☐	☐
8. ¿Qué importancia tiene para usted la capacidad de reconocer una melodía familiar (p.ej. una canción, un/a cantante, una pieza musical)?	☐	☐	☐	☐	☐	☐
9. ¿Qué importancia tiene para usted la capacidad de valorar la calidad de una interpretación musical (p.ej. cuando alguien canta o toca un instrumento musical)?	☐	☐	☐	☐	☐	☐
10. ¿Qué importancia tiene para usted tener la sensación de que escucha música igual que otras personas?	☐	☐	☐	☐	☐	☐
11. ¿Qué importancia tiene para usted escuchar la música afinada?	☐	☐	☐	☐	☐	☐
**DEDICACIÓN A LA MÚSICA**						
12. ¿Qué importancia tiene para usted disfrutar de la música en entornos ruidosos si no hay referencias visuales (p.ej. en una fiesta, en un restaurante o en el coche con el ruido del motor o de la calle de fondo)?	☐	☐	☐	☐	☐	☐
13. ¿Qué importancia tiene para usted disfrutar de la música en la televisión, en el reproductor de DVD o en el ordenador?	☐	☐	☐	☐	☐	☐
14. ¿Qué importancia tiene para usted disfrutar de la música de fondo durante la práctica de actividades como la lectura, la pintura, la jardinería, el ejercicio físico, etc. o mientras se relaja?	☐	☐	☐	☐	☐	☐
15. ¿Qué importancia tiene para usted escuchar música al viajar (p.ej. en el coche)?	☐	☐	☐	☐	☐	☐
16. ¿Qué importancia tiene para usted escuchar música nueva (es decir, música que no ha escuchado antes)?	☐	☐	☐	☐	☐	☐
17. ¿Qué importancia tiene para usted asistir a eventos musicales públicos (p.ej. musicales, conciertos o festivales de música)?	☐	☐	☐	☐	☐	☐
18. ¿Qué importancia tienen para usted cantar, tocar un instrumento musical o silbar?	☐	☐	☐	☐	☐	☐

NOMBRE____ FECHA_______________

INTERESES MUSICALES

1 - ¿Te gusta escuchar música? NO☐ YA NO, ANTES☐ A VECES ☐ SÍ☐

2 - ¿Te gusta cantar? NO☐ YA NO, ANTES☐ A VECES ☐ SÍ☐

3 - ¿Tocas algún instrumento musical? NO☐ YA NO, ANTES☐ A VECES ☐ SÍ☐

4 - ¿Te gusta bailar? NO☐ YA NO, ANTES☐ A VECES ☐ SÍ☐

5 - ¿Te resulta fácil bailar al ritmo de la música? NO☐ YA NO, ANTES☐ A VECES ☐ SÍ☐

6 - ¿Te resulta fácil aprender una nueva melodía? NO☐ YA NO, ANTES☐ A VECES ☐ SÍ☐

7 - ¿Afecta la música a tu estado de ánimo? NO☐ YA NO, ANTES☐ A VECES ☐ SÍ☐

PERFIL MUSICAL

8. ¿Cuántos días por semana escuchas música?

1	2	3	4	5	6	7
☐	☐	☐	☐	☐	☐	☐

9. ¿Con qué frecuencia <u>escuchas música</u> en las siguientes situaciones?

	Nunca (1%)	*Raras veces* (12%)	*De vez en cuando (25%)*	*Generalmente* (50%)	*Frecuentemente* (75%)	*Casi* siempre (87%)	*Siempre* (99%)
9.1.Cuando estoy sol@ en casa	1☐	2☐	3☐	4☐	5☐	6☐	7☐
9.2.Cuando estoy acompañad@ en casa	1☐	2☐	3☐	4☐	5☐	6☐	7☐
9.3.Cuando estoy con amig@s	1☐	2☐	3☐	4☐	5☐	6☐	7☐
9.4.Cuando me voy a dormir	1☐	2☐	3☐	4☐	5☐	6☐	7☐
9.5.Para relajarme	1☐	2☐	3☐	4☐	5☐	6☐	7☐
9.6.Cuando hago deporte	1☐	2☐	3☐	4☐	5☐	6☐	7☐
9.7.Mientras viajo (yendo al colegio…)	1☐	2☐	3☐	4☐	5☐	6☐	7☐
9.8.Mientras estudio	1☐	2☐	3☐	4☐	5☐	6☐	7☐

10. ¿Con qué frecuencia cantas?

Nunca (1%)	*Raras veces* (12%)	*De vez en cuando (25%)*	*Generalmente* (50%)	*Frecuentemente* (75%)	*Casi todo* el tiempo (87%)	*Todo el tiempo* (99%)
1☐	2☐	3☐	4☐	5☐	6☐	7☐

11. ¿Con qué frecuencia cantas en las siguientes situaciones?

	Nunca (1%)	*Raras veces* (12%)	*De vez en cuando* (25%)	*Generalmente* (50%)	*Frecuentemente* (75%)	*Casi* siempre (87%)	*Siempre* (99%)
11.1.Cuando estoy sol@ en casa	1☐	2☐	3☐	4☐	5☐	6☐	7☐
11.2.Cuando estoy acompañad@ en casa	1☐	2☐	3☐	4☐	5☐	6☐	7☐
11.3.Cuando estoy con amig@s	1☐	2☐	3☐	4☐	5☐	6☐	7☐
11.4.Delante de otros	1☐	2☐	3☐	4☐	5☐	6☐	7☐
11.5.Delante de una grabación	1☐	2☐	3☐	4☐	5☐	6☐	7☐

PAPEL DE LA MÚSICA

12. ¿Qué importancia tiene para ti la música en tu vida?

Nada importante *Muy importante*

1☐ 2☐ 3☐ 4☐ 5☐ 6☐ 7☐

ACTIVIDADES RELACIONADAS CON LA MÚSICA

13. ¿Realizas alguna actividad musical (aparte de las clases de música en el colegio)? Por ejemplo: tocar la guitarra, el piano, teatro musical, tocar en un grupo musical…

SI ☐
NO ☐

Si has contestado que sí, ¿qué actividad realizas? ¿Y durante cuánto tiempo llevas haciéndola?

13.1. ☐ Grupo musical ___________años/meses
13.2. ☐ Coro musical ___________años/meses
13.3. ☐ Clases particulares de algún instrumento o de voz ___________años/meses
13.4. ☐ Tocar por diversión yo sol@ ___________años/meses
13.5. ☐ Tocar por diversión con amig@s ___________años/meses
13.6. ☐ Otra: ____ ___________años/meses

- Si la actividad no la realizas actualmente, ¿la has realizado hace tiempo?
